# Pseudocapacitive Effects of *N*-Doped Carbon Nanotube Electrodes in Supercapacitors

**DOI:** 10.3390/ma5071258

**Published:** 2012-07-19

**Authors:** Young Soo Yun, Hyun Ho Park, Hyoung-Joon Jin

**Affiliations:** Department of Polymer Science and Engineering, Inha University, Incheon 402-751, Korea; E-Mails: ysyun@inha.edu (Y.S.Y.); hhpark@inha.edu (H.H.P.)

**Keywords:** pseudocapacitance, supercapacitor, carbon nanotubes

## Abstract

Nitrogen- and micropore-containing carbon nanotubes (NMCNTs) were prepared by carbonization of nitrogen-enriched, polymer-coated carbon nanotubes (CNTs), and the electrochemical performances of the NMCNTs with different heteroatom contents were investigated. NMCNTs-700 containing 9.1 wt% nitrogen atoms had a capacitance of 190.8 F/g, which was much higher than that of pristine CNTs (48.4 F/g), despite the similar surface area of the two CNTs, and was also higher than that of activated CNTs (151.7 F/g) with a surface area of 778 m^2^/g and a nitrogen atom content of 1.2 wt%. These results showed that pseudocapacitive effects play an important role in the electrochemical performance of supercapacitor electrodes.

## 1. Introduction

Carbon nanotubes (CNTs) have been studied extensively in various fields due to their excellent electrical and mechanical properties and unique one-dimensional geometry [1,2,3,4,5,6]. However, the specific capacitance values of CNTs as an electrode for supercapacitors are much lower than those of activated carbons because of the relatively low surface areas and deficiency of micropores, which are advantageous in electrical double layer capacitance. The electrochemical performance of CNTs can be increased by introducing heteroatoms [7,8,9,10]. Heteroatoms on carbon surfaces dramatically enhance specific capacitance values of carbon materials by pseudocapacitive effects [7,8,9,10]. Even nonporous carbon materials containing nitrogen atom exhibited a specific capacitance of 198 F/g in aqueous solvent [8]. Surface modification is used to incorporate electroactive heteroatoms on CNTs [11,12,13,14]. However, there are limitations with some fussy approaches or harsh reaction conditions. Incorporating a heteroatom-enriched carbon layer on the CNT surface can be a viable alternative for CNTs with both heteroatoms and sufficient micropores. Many polymers containing heteroatoms in their molecular structure have been used to prepare heteroatom-enriched carbon materials with high surface area, numerous micropores and electroactive heteroatoms [7,8,9,15,16].

In this study, nitrogen- and micropore-containing CNTs (NMCNTs) were prepared by carbonization of nitrogen-enriched, polymer-coated CNTs. The NMCNTs had different nitrogen atom contents according to the carbonization condition. The nitrogen content was inversely proportional to the carbonization temperature. In contrast, the specific surface areas of all the NMCNTs were similar with each other. These results were used to investigate the contribution of the nitrogen atoms to the electrochemical performance of the NMCNTs. The NMCNTs had numerous micropores, which became dramatically more numerous after activation by the chemical agent, KOH, while most of the nitrogen atoms of the activated NMCNTs were removed by the activation. These results were used to investigate the effect of the micropores on the electrochemical performance of the NMCNTs.

## 2. Results and Discussion

**Figure 1:**
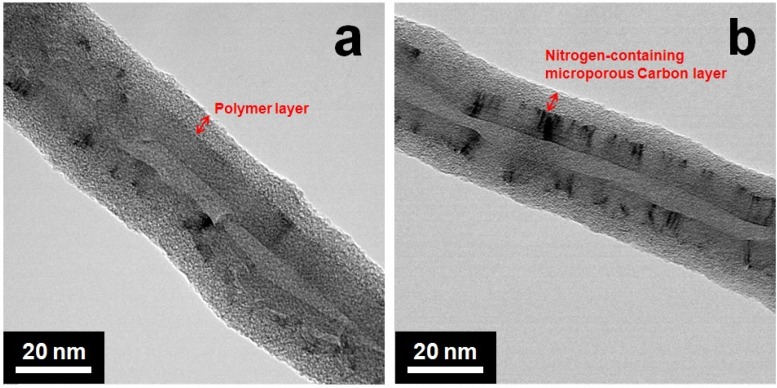
TEM images of (**a**) polymer-coated carbon nanotubes (CNTs); and (**b**) nitrogen- and micropore-containing CNTs (NMCNTs).

Figure 1 shows the morphologies of the polymer-coated CNTs and NMCNTs. A polymer layer of about 5–7 nm thickness that was coated on the surface of the CNTs was maintained after carbonization.

**Figure 2:**
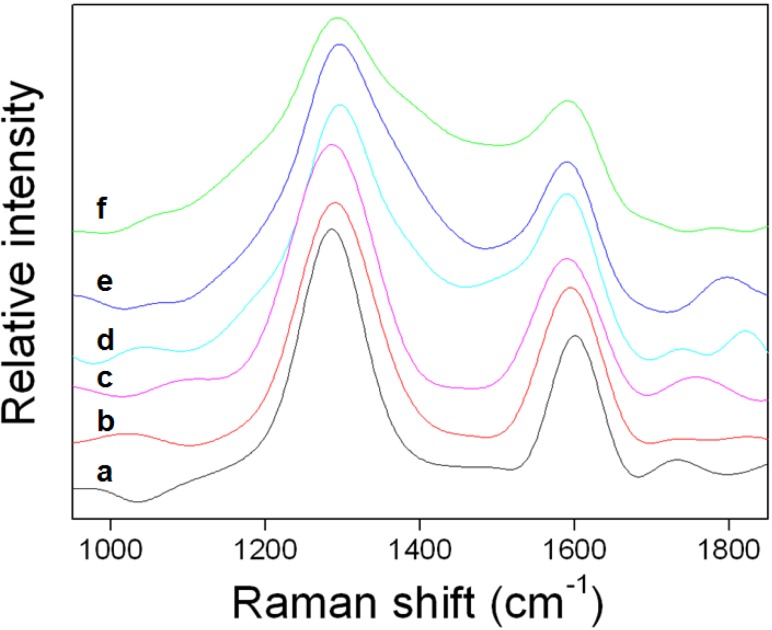
Raman spectra of (**a**) pristine CNTs; (**b**) activated NMCNTs; (**c**) NMCNTs-1000; (**d**) NMCNTs-900; (**e**) NMCNTs-800; and (**f**) NMCNTs-700.

The Raman spectrum of the pristine CNTs exhibited two Raman bands at ~1601 cm^−1^ due to the structure of the sp^2^ hexagonal carbon bond network, known as the “G” peak, and at ~1420 cm^−1^, designated as “D”, due to disorder in the graphene layers of the sp^3^ carbon bond (Figure 2). The ratio of the intensities of the G and D bands (I_G_/I_D_) of the pristine CNTs was 0.56 and the Raman spectra of the NMCNTs and activated NMCNTs also exhibited two Raman bands at G peak and D peak, which confirmed the successful conversion of the polymer layer to the carbon layer.

The textural properties of the pristine CNTs, NMCNTs and activated NMCNTs are shown in Table 1. The pristine CNTs had a surface area of 218 m^2^/g, most of which was induced by the mesopores. In contrast, the activated NMCNTs and NMCNTs had many micropores and mesopores, which revealed the microporous nature of the carbon layer on the surface of the NMCNTs. However, the surface areas of the NMCNTs were similar to that of the pristine CNTs because the carbon layers blocked the holes in the mesoporous end of the CNTs.

**Table 1:** Textural properties of the pristine carbon nanotubes (CNTs), activated CNTs and nitrogen- and micropore-containing CNTs (NMCNTs).

Samples	S_BET_ (m^2^/g)	S_mic_ (m^2^/g)	S_meso_ (m^2^/g)	V_total_ (cm^3^/g)
Pristine CNTs	218	19	199	0.85
activated CNTs	778	197	518	0.54
NMCNTs-700	200	126	74	0.31
NMCNTs-800	218	130	88	0.39
NMCNTs-900	237	141	96	0.45
NMCNTs-1000	204	118	86	0.43

**Figure 3:**
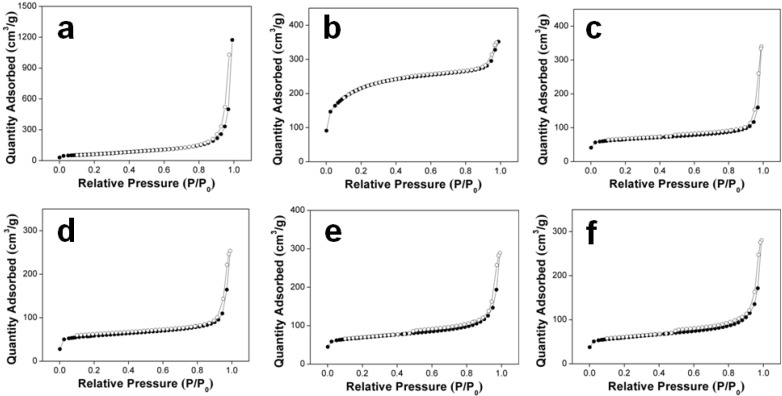
Nitrogen adsorption and desorption isotherms of (**a**) pristine CNTs; (**b**) activated NMCNT; (**c**) NMCNTs-700; (**d**) NMCNTs-800; (**e**) NMCNTs-900; and (**f**) NMCNTs-1000.

Figure 3 shows the nitrogen adsorption and desorption isotherms of the samples. The isotherm curve of the pristine CNTs exhibited an IUPAC-IV shape, which suggested a mesoporous structure. In contrast, the isotherms of the NMCNTs and activated NMCNTs exhibited IUPAC Type-I and -IV hybrid shapes, respectively, which suggested dual microporous and mesoporous structures.

**Figure 4:**
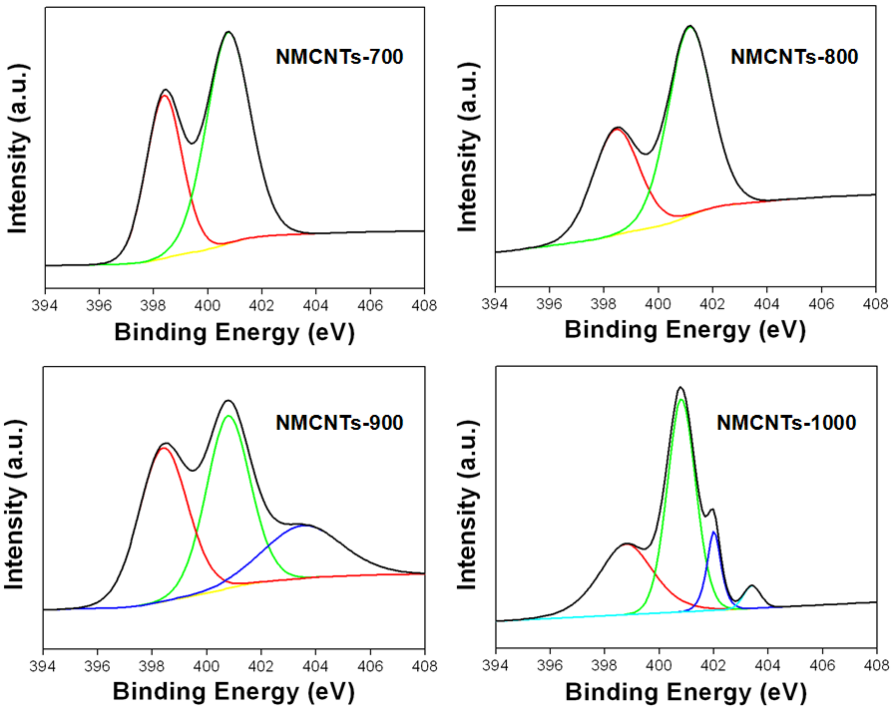
X-ray photoelectron spectroscopy (XPS) N 1s spectra of NMCNTs-700, -800, -900 and -1000.

Figure 4 shows the XPS N 1s spectra of the NMCNTs. The NMCNTs contained the number and kind of nitrogen species, and the composition of the nitrogen species was changed according to the carbonization condition.

Table 2 shows further specific information related to atomic contents, and relative surface concentrations of the nitrogen species obtained by fitting the N 1s core level XPS spectra. NMCNTs-700 had numerous nitrogen atoms, with a content of 9.1 at.%, which were formed of electroactive pyridinic-N (N-6) and pyrrolic-N (N-5). As a carbonization temperature was increased, the nitrogen contents of the NMCNTs were decreased, and the samples carbonized above 900 °C had quaternary N (N-Q) and pyridinic oxide (N-X), as well as pyridinic-N (N-6) and pyrrolic-N (N-5) [17,18]. However, the activated CNTs lost most of their nitrogen atoms but gained some oxygen atoms.

**Table 2:** Carbon, nitrogen and oxygen contents, and relative surface concentrations of nitrogen species obtained by fitting the N1s core level X-ray photoelectron spectroscopy (XPS) spectra.

Samples	C (at.%)	N (at.%)	O (at.%)	N-6 (%)	N-5 (%)	N-Q (%)	N-X (%)
pristine CNTs	97.6	–	2.4	–	–	–	–
activated CNTs	92.5	1.2	6.3	–	–	–	–
NMCNTs-700	85.9	9.1	5.0	38.5	61.5	–	–
NMCNTs-800	87.1	8.4	4.5	37.1	62.9	–	–
NMCNTs-900	90.5	6.2	3.3	39.0	39.6	–	21.4
NMCNTs-1000	92.6	4.1	3.3	34.0	52.6	10.0	3.4

**Figure 5:**
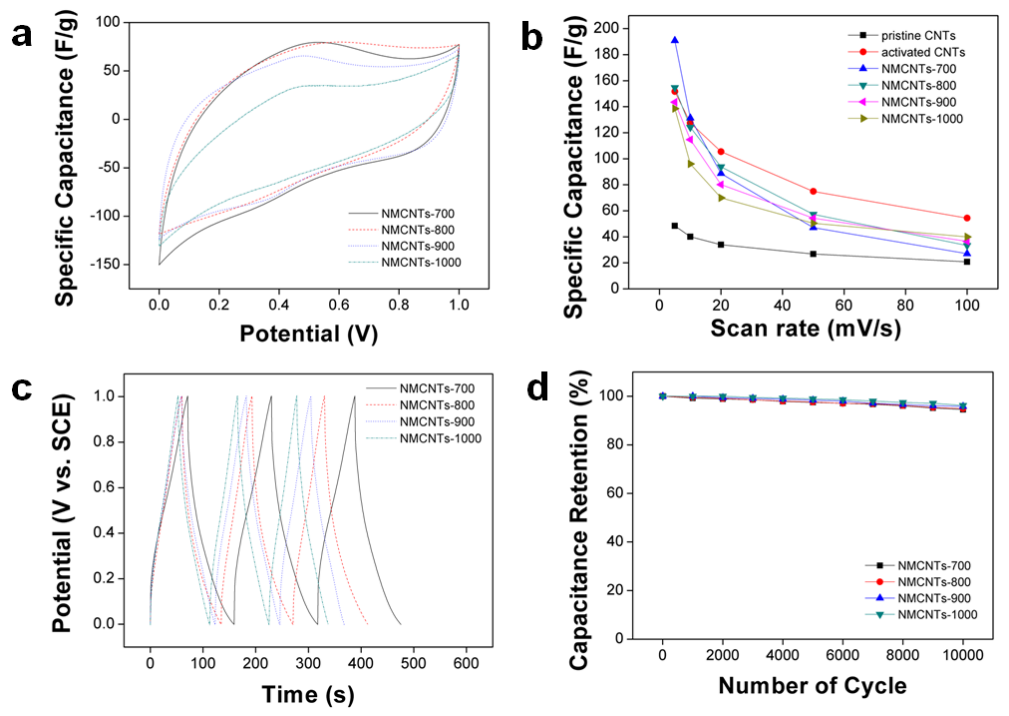
(**a**) Cyclic voltammograms of the NMCNTs at a scan rate of 10 mV/s over a potential range of 0 to 1 V in a 1M H_2_SO_4_ aqueous solution; (**b**) capacitance of the cells calculated from cyclic voltammograms at different scan rates; **(c**) galvanostatic charge/discharge curves of the NMCNTs in the potential window of 0 to 1 V at a current density of 1 A/g; and (**d**) variations of the specific capacitance of NMCNTs as a function of the cycle number measured at 100 mV/s in a 1 M H_2_SO_4_ aqueous solution.

The electrochemical performances of the pristine CNTs, activated CNTs and NMCNTs were analyzed using CV and galvanostatic charge/discharge (Figure 5). The CV curves of the NMCNTs were similar to each other and exhibited a humped appearance, due to the redox reactions related to the heteroatom functionalities of the materials (Figure 5a). The result suggested that the pseudocapacitive reaction contributed to the capacitance of the NMCNTs. The pristine CNTs had a capacitance of 48.4 F/g at a scan rate of 5 mV/s, compared to 190.8, 154.7, 143.5 and 138.6 F/g for NMCNTs-700, -800, -900 and -1000, respectively. Despite the similar surface area of the pristine CNTs and NMCNTs, their capacitances were quite different, which suggested that the pseudocapacitive effects of the nitrogen groups play an important role in the electrochemical performance of electrode materials. The capacitance of the samples increased with increasing number of nitrogen atoms. In addition, the capacitance of the activated CNTs, which lost most of their nitrogen atoms during the activation process, at 151.7 F/g was much higher than that of the pristine CNTs. This result suggested that the surface areas of the sample also play an important role in the electrochemical performance of the electrode materials. The activated CNTs with a surface area of 778 m^2^/g had a 3-fold higher capacitance than that of the pristine CNTs with a surface area of 218 m^2^/g. However, although the surface area of NMCNTs-700 was similar to that of the pristine CNTs, the capacitance of NMCNTs-700 was higher than that of the activated CNTs at low scan rates. The result demonstrated the importance of the heteroatom in enhancing the capacitance of electrode materials. However, the capacitance of the NMCNTs at higher scan rates dropped sharply and that of the activated CNTs at scan rates above 10 mV/s was even larger than that of the NMCNTs. This result was attributed to the fact that the pseudocapacitive behavior of heteroatoms had an insufficient effect on the condition at higher scan rates. Figure 5c shows the galvanostatic charge/discharge curves of the NMCNTs. A sharp IR drop was observed in NMCNTs-700 due to the electroresistivity caused by numerous heteroatoms and the IR drop was decreased by the reduction of heteroatoms in the samples. The capacitance retention of all the samples was very good (Figure 5d) with a capacitance loss of only 5.5% over 10,000 cycles for NMCNTs-700, which demonstrated the good physical stabilities of the CNTs.

## 3. Experimental Section

### 3.1. Materials

Multiwalled CNTs (purity of 96% based on thermogravimetric analysis, a diameter of 10 to 15 nm and a length of about 20 µm, supplied by Hanwha Nanotech Corp., Incheon, Korea) were produced through thermal chemical vapor deposition. An aniline monomer was purchased from DC Chemical Co., Ltd. (Seoul, Korea), and ammonium persulfate (APS, 98%) was purchased from DAEJUNG Chemicals and Materials Co. (Gyeonggi-Do, Korea). Sodium dodecyl sulfate (SDS, SIGMA, Saint Louis, MO, USA), acetic acid (OCI Company Ltd., Seoul, Korea), KOH (OCI Company Ltd., Seoul, Korea) and all other organic solvents were used without further purification.

### 3.2. Preparation of Polymer-Coated CNTs

The polymer-coated CNTs were prepared via *in situ* oxidative polymerization in the presence of a CNT suspension and aniline monomer. First, 200 mg of CNTs and 60 mg of SDS were added into 200 mL of 3 M acetic acid solution. After 2 mL of aniline monomer was added into the previously prepared solution, the solution was ultrasonicated for 24 h. Afterwards, 2 g of APS as an oxidant in 50 mL of 3 M acetic acid was rapidly added into the above mixed solution that was maintained at room temperature for 12 h. Finally, the polymer-coated CNTs were washed with ethanol and deionized water repeatedly, and then dried in an oven 60 °C.

### 3.3. Preparation of the NMCNTs and the Activated NMCNTs

The polymer-coated CNTs were carbonized in a horizontal furnace under 150 cc/min of pure nitrogen gas atmosphere at a heating rate of 10 °C/min from room temperature to the final temperatures of 700, 800, 900 and 1000 °C, and then maintained at these temperatures for 2 h. The carbonized samples were termed NMCNTs-700, -800, -900, and -1000, respectively. The polymer-coated NMCNTs with KOH powder were ground thoroughly and mixed at a 1:1 weight ratio. The well-ground powder was activated in a horizontal furnace under 150 cc/min of pure nitrogen gas at a heating rate of 10 °C/min from room temperature to the final temperature of 800 °C, and then maintained at this temperature for 2 h. The activated sample was termed activated NMCNTs.

### 3.4. Characterization

The surface of the samples was confirmed by examining the internal structure by using transmission electron microscopy (TEM, CM200, Philips, New York, NY, USA) at an accelerating voltage of 100 kV. Raman spectra were collected with a 1064 nm laser source on a Raman spectrometer (RFS 100/S, Bruker, Karlsruhe, Germany) from 2500 cm^−1^ to 800 cm^−1^ at 80 mV. Wide-angle X-ray diffraction (WAXD, Rigaku, DMAX-2500, Tokyo, Japan) was used to characterize the samples through a diffractometer with reflection geometry and CuKα radiation (wavelength λ = 0.154 nm) operated at 40 kV and 100 mA. The data were collected over a range of scattering angles (2θ) from 5° to 60° with a scanning rate of 1/min. The chemical state and surface composition of the samples were measured by using X-ray photoelectron spectroscopy (XPS, AXIS-HIS, Kratos Analytical, Hadano, Japan). The XPS spectra were recorded using a dual-chromatic MgKα X-ray source at 1500 eV. High-resolution XPS spectra were recorded in 0.05 eV steps with a pass energy of 20 eV. The porous properties of the samples were analyzed from the nitrogen adsorption and desorption isotherms obtained using a surface area and porosimetry analyzer (ASAP 2020, Micromeritics, Norcross, GA, USA) at −196 °C. The Brunauer–Emmett–Teller (BET) surface areas (S_BET_) were calculated using the BET theory. The micropore surface area (S_mic_) and micropore volume were obtained using t-plot theory. The mesopore surface area (S_meso_) and mesopore volume were calculated in accordance with the Barrett–Joyner–Halenda (BJH) theory.

### 3.5. Electrochemical Measurements

The working electrodes were prepared as follows. The electroactive materials and polytetrafluoroethylene as a binder were mixed at a mass ratio of 90:10, dissolved in ethanol, coated onto a nickel mesh substrate (0.785 cm^2^) with a spatula, and dried at 110 °C for several hours in an oven. The amount of electroactive materials was kept constant for each electrode. Each electrode contained about 2.5 mg of the electroactive materials. All electrochemical measurements were performed in a three-electrode system. The nickel mesh containing the electroactive materials, platinum plate and saturated KCl were used as the working, counter and reference electrodes, respectively. The measurements were carried out in a 1M H_2_SO_4_ acidic solution at room temperature. Then the cyclic voltammetry (CV), galvanostatic charge/discharge results were measured by a potentiostat/galvanostat (PGSTAT302N, Autolab). CV tests were performed between 0 and 1 V (*vs*. SCE) at different scan rates. The galvanostatic charge/discharge tests were measured in the potential of 0 to 1 V (*vs*. SCE) at a current density of 1 A/g.

## 4. Conclusions

We successfully prepared NMCNTs by carbonization of nitrogen-enriched, polymer-coated CNTs. NMCNTs-700, -800, -900 and -1000, which were carbonized under the indicated carbonization temperatures, had different nitrogen contents of 9.1, 8.4, 6.2 and 4.1 at.%, respectively. In addition, the NMCNTs had numerous micropores, while the surface areas of the NMCNTs were similar to those of the pristine CNTs. The capacitance of NMCNTs-700 (190.8 F/g) was much higher than that of the pristine CNTs (48.4 F/g) and was even higher than that of activated CNTs (151.7 F/g) with a surface area of 778 m^2^/g at a scan rate of 5 mV/s. These results showed that the pseudocapacitive effects induced by the nitrogen groups play an important role in the electrochemical performance of electrode materials. The capacitance retention was very high with a capacitance loss of only 5.5% over 10,000 cycles for NMCNTs-700.
